# Genetics at Belyaev Conference – 2017: introductory note

**DOI:** 10.1186/s12863-017-0577-4

**Published:** 2017-12-28

**Authors:** Yuriy L. Orlov, Ancha V. Baranova, Tatiana V. Tatarinova, Nikolay A. Kolchanov

**Affiliations:** 1 Institute of Cytology and Genetics SB RAS, Novosibirsk, Russia; 2 Novosibirsk State University, Novosibirsk, Russia; 3 Research Centre of Medical Genetics, Moscow, Russia; 4 George Mason University, Fairfax, VA USA; 5 University of LaVerne, Los Angeles, CA USA

This thematic issue of *BMC Genetics* continues the series of BioMed Central special post-conference issues presenting materials from the conferences on bioinformatics and systems biology BGRS\SB (Bioinformatics of Genome Regulation and Structure\Systems Biology) annually held in Novosibirsk. Here we present the papers discussed at and Belyaev Conference-2017 “Belyaev Readings - 2017” (BR-2017). The Year 2017 marks the 100-th anniversary since birth of Full Member of the USSR Academy of Sciences, Professor Dmitry K. Belyaev (1917–1985), an outstanding scientist, evolutionist and geneticist. In view of this memorable date, the Institute of Cytology and Genetics of the Siberian Branch of the Russian Academy of Sciences (ICG SB RAS) held international Belyaev Conference on Genetics and Evolution (Novosibirsk, August 7–10, 2017 - http://conf.bionet.nsc.ru/belyaev100/en).

Previously published special issues of *BMC Genetics, BMC Genomics* and *BMC Evolutionary Biology* covered the proceeding of BGRS\SB-2016 conference and SBB-2015 School in Novosibirsk [1–5] as well as BGRS\SB-2014 event (https://bmcgenomics.biomedcentral.com/articles/supplements/volume-15-supplement-12). First BMC *Genetics* special issue paper in the area of genetics was presented at the BGRS\SB-2014 conference series in Novosibirsk [6].

The memorial Belyaev conference-2017 continued a tradition of BGRS\SB series on bioinformatics and systems biology. In 2017 “Vavilov Journal of Selection and Breeding” published a series of memoirs publications about Prof. Belyaev (http://vavilov.elpub.ru/jour/issue/view/32/showToc). In particular, the article by Prof. V. K. Shumny [7] walks the reader along milestones of Belyaev’s life, while other publications discuss critical influence of Belyaev’s work on the theory of evolution and domestication [8, 9]: N. K. Popova discussed evolution of the brain in the process of domestication [10] and P. D. Polly presented the review of the connections of morphometry and evolutionary theory fields [11].

Genetics-related works discussed at Belyaev conference-2017 are collated in present issue of *BMC Genetics.*

The work by Triska and co-authors ([12], this issue) describes genetic history of the migration of humans throughout the Europe and North Asia. International team from more than a dozen of different institutions genotyped and analyzed more than a thousand individuals from 30 ethnic populations residing in regions which span from Baltic Sea to Baikal Lake. The dense sampling allowed much needed detailed description of population structure and provided critical insights into genomic history of European - Asian interface, thus, significantly increasing quality of genetic coverage for modern populations in region of North Eurasia.

The paper by Ranajit Das and Priyanka Upadhyai ([13], this issue) continues the theme of human genetics by analyzing populations of South Asia presented in the dataset released by 1000 Genomes Project. Das and Upadhyai dissected complex history of population dispersal and gene flow in the Indian subcontinent by employing the Geographic Population Structure (GPS) tool to define contributions of five South Asian populations, Punjabi, Gujarati, Tamil, Telugu and Bengali into many recent migrant populations sampled elsewhere.

Nikolay S. Yudin and colleagues ([14], this issue) continue the topic of population genetics considering adaptation to cold environment. A compendium combining mammalian genes evolved to adapt to cold environment was analyzing an intersection of positively selected genes from six Arctic and Antarctic species. This compendium lists about four hundreds genes that have been positively selected in at least two species. However, no positively selected genes related to cold adaptation were common for all the species, thus, indicating the complexity of the mechanisms allowing adapting to cold.

The paper by Fedorova et al. ([15], this issue) explores *NETO2* gene upregulation along with deregulation of eight epithelial-mesenchymal transition-related genes in colorectal cancer.

Alexei N. Korablev and colleagues ([16], this issue) describe CRISPR/Cas9 genome editing approach for generation of megabase-scale deletions, inversions and duplications in mice. Copy Number Variation (CNV) of the human *CNTN6* gene, which encodes the contactin-6 protein, is responsible for severe neurodevelopmental impairments, often in combination with facial dysmorphias. Mice carrying megabase-scale deletions, duplications, and inversions involving the full-sized *Cntn6* gene were thoroughly characterized.

Valeriya Vavilova and co-authors ([17], this issue) studies DEP1 gene variants in wheats with either normal or compact spike shape from eight accessions which belong to four wheat species, *T. monococcum, T. durum, T. compactum,* and *T. spelta*, and showed that DEP1 does not directly participate in the control of the spike architecture.

Follow-on series of related works in the areas of classical and medical genomics, genetics, and plant biology discussed at “Belyaev conference – 2017” and other related meetings in Novosibirsk and Moscow are published in Special Issues of *BMC Evolutionary Biology, BMC Plant Biology, BMC Genomics, BMC Medical Genomics* and *BMC Neuroscience.* The Proceedings of the conference are available at http://conf.bionet.nsc.ru/belyaev100/en/ and http://conf.bionet.nsc.ru/belyaev100/wp-content/uploads/sites/14/2017/01/BELYAEV_conf_2_08_2017.pdf.

The readers are welcome to visit Novosibirsk at the time of next XI-the BGRS\SB-2018 conference on August 20-28th in 2018.

## References

[1] Orlov YL, Baranova AV, Markel AL (2016). Computational models in genetics at BGRS\SB-2016: introductory note. BMC Genet.

[2] Orlov YL, Baranova AV, Hofestädt R, Kolchanov NA (2016). Computational genomics at BGRS\SB-2016: introductory note. BMC Genomics.

[3] Baranova AV, Orlov YL (2016). The papers presented at 7th young scientists school "systems biology and bioinformatics" (SBB'15): introductory note. BMC Genet.

[4] Orlov YL, Hofestädt RM, Kolchanov NA (2015). Introductory note for BGRS\SB-2014 special issue. J Bioinforma Comput Biol.

[5] Baranova AV, Orlov YL. Evolutionary biology at BGRS\SB-2016. BMC Evol Biol. 2017;17(Suppl 1):21). doi:10.1186/s12862-016-0869-8. 10.1186/s12862-016-0869-8 PMC5333185 28251876

[6] Redina OE, Smolenskaya SE, Klimov LO, Markel AL (2015). Candidate genes in quantitative trait loci associated with absolute and relative kidney weight in rats with inherited stress induced arterial hypertension. BMC Genet.

[7] Shumny VK. To the centenary of the birth of outstanding evolutionist Dmitri Konstantinovich Belyaev. Vavilovskii Zhurnal Genetiki i Selektsii = Vavilov. J Genet Breed. 2017;21(4):387–91. doi:10.18699/VJ17.256 (In Russian).

[8] Trut LN, Kharlamova AV, Vladimirova AV, Herbeck YE. On selection of foxes for enhanced aggressiveness and its correlated implications. Vavilovskii Zhurnal Genetiki i Selektsii = Vavilov. J Genet Breed. 2017;21(4):392–401. doi:10.18699/VJ17.257 (In Russian).

[9] Wilkins AS. Revisiting two hypotheses on the “domestication syndrome” in light of genomic data. Vavilovskii Zhurnal Genetiki i Selektsii = Vavilov. J Genet Breed. 2017;21(4):435–42. doi:10.18699/VJ17.262.

[10] Popova NK. The domestication and the brain: forty years after. Vavilovskii Zhurnal Genetiki i Selektsii = Vavilov. J Genet Breed. 2017;21(4):414–20. doi:10.18699/VJ17.259 (In Russian).

[11] Polly PD. Morphometrics and evolution: the challenge of crossing rugged phenotypic landscapes with straight paths. Vavilovskii Zhurnal Genetiki i Selektsii = Vavilov. J Genet Breed. 2017;21(4):452–61. doi:10.18699/vJ17.264.

[12] Triska P, Chekanov N, Stepanov V, Khusnutdinova EK, Kumar GPA, Akhmetova V, Babalyan K, Boulygina E, Kharkov V, Gubina M, Khidiyatova I, Khitrinskaya I, Khrameeva EE, Khusainova R, Konovalova N, Litvinov S, Marusin A, Mazur AM, Puzyrev V, Ivanoshchuk D, Spiridonova M, Teslyuk A, Tsygankova S, Triska M, Trofimova N, Vajda E, Balanovsky O, Baranova A, Skryabin K, Tatarinova TV, Prokhortchouk E. Between Lake Baikal and the Baltic Sea: genomic history of the Gateway to Europe. BMC Genetics. 2017;18(Suppl 1):S2. doi:10.1186/s12863-017-0578-3 10.1186/s12863-017-0578-3 PMC5751809 29297395

[13] Das Rt, Upadhyai P. Application of Geographical Population Structure (GPS) algorithm for biogeographical analyses of populations with complex ancestries: a case study of South Asians from 1000 Genomes project. BMC Genetics. 2017;17(Suppl 1):S3. doi:10.1186/s12863-017-0579-2 10.1186/s12863-017-0579-2 PMC5751663 29297311

[14] Yudin NS, Larkin DM, Ignatieva EV. A compendium and functional characterization of mammalian genes involved in adaptation to cold environment. BMC Genetics. 2017;18(Suppl 1):S4. doi:10.1186/s12863-017-0580-9 10.1186/s12863-017-0580-9 PMC5751660 29297313

[15] Fedorova MS, Snezhkina AV, Pudova EA, Abramov IS, Lipatova AV, Kharitonov SL, Sadritdinova AF, Nyushko KM, Klimina KM, Belyakov MM, Slavnova EN, Melnikova NV, Chernichenko MA, Sidorov DV, Kaprin AD, Alekseev BY, Dmitriev AA, Kudryavtseva AV. Upregulation of *NETO2* gene in colorectal cancer. BMC Genetics. 2017;18(Suppl 1):S5. doi:10.1186/s12863-017-0581-8 10.1186/s12863-017-0581-8 PMC5751543 29297384

[16] Korablev AN, Serova IA, Serov OL. Generation of megabase-scale deletions, inversions and duplications involving the *Contactin-6* gene in mice by CRISPR/Cas9 technology BMC Genetics. 2017;18(Suppl 1):S6. doi:10.1186/s12863-017-0582-7 10.1186/s12863-017-0582-7 PMC5751523 29297312

[17] Vavilova V, Konopatskaia I, Kuznetsova AE, Blinov A, Goncharov NP. *DEP1* gene in wheat species with normal, compactoid and compact spikes. BMC Genetics. 2017;18(Suppl 1):S7. doi:10.1186/s12863-017-0583-6 10.1186/s12863-017-0583-6 PMC5751790 29297308

